# Non-operative treatment or volar locking plate fixation for dorsally displaced distal radius fractures in patients over 70 years – a three year follow-up of a randomized controlled trial

**DOI:** 10.1186/s12891-022-05394-7

**Published:** 2022-05-12

**Authors:** Hanna Südow, Sara Severin, Maria Wilcke, Jenny Saving, Olof Sköldenberg, Cecilia Mellstrand Navarro

**Affiliations:** 1 Department of Clinical Science and Education, Karolinska Institute, Södersjukhuset, SE-118 83 Stockholm, Sweden; 2 Department of Orthopedics, Södersjukhuset Hospital, Stockholm, Sweden; 3 Karolinska Institute, Department of Clinical Science, Danderyd Hospital, Stockholm, Sweden; 4 Department of Orthopedics, Danderyd University Hospital Corp, Stockholm, Sweden; 5 Department of Hand Surgery, Södersjukhuset Hospital, Stockholm, Sweden; 6 Capio Artro Clinic, Stockholm, Sweden

**Keywords:** Distal radius fracture, Geriatric fracture, Volar locking plate, Non-operative treatment, Fracture fixation, Aged, Quality of life

## Abstract

**Background:**

Surgical treatment of displaced distal radius fractures (DRF) in older patients has increased, despite lacking evidence of its superiority over non-operative treatment. How treatment choice affects these patients after the initial 12-month period remains unknown. This study presents a clinical and radiographic follow up at an average of 3 years after treatment in the context of a randomized clinical trial comparing outcomes in patients aged ≥70 years, with a dorsally displaced distal radius fracture treated either surgically with volar locking plate or non-operatively.

**Methods:**

Between 2009 and 2017, 140 patients aged ≥70 years with dorsally displaced DRF were randomized to surgery with volar locking plate (VLP) or non-operative treatment. At an average of 3 years after inclusion the participants were invited to an additional follow-up. The primary outcome was Patient Rated Wrist Evaluation (PRWE). Secondary outcomes included additional Patient Reported Outcome Measures (PROM), grip strength, range of motion, complications and radiological results.

**Results:**

Sixty six patients were available for a 3 year follow-up, 33 in the non-operatively treated group and 33 in the VLP-group. The mean age at injury was 77 years. At 3 years the median PRWE was better (0 points) in the VLP-group than in the non-operative treatment group (9 points) *p*-value: 0.027. No statistically significant difference was found in Disabilities of the Arm, Hand, and Shoulder (DASH), EuroQol 5 Dimensions (EQ-5D) or grip strength. Total arc of range of motion was larger in the operatively treated group. No significant difference in osteoarthritis was found. Both groups had regained grip strength. The complication rate was similar. Outcomes improved from the 1 year to the 3 year follow-up.

**Conclusions:**

Surgery with volar locking plate gave less long-term disability compared to non-operative treatment for severely displaced distal radius fractures in patients aged ≥70 years. Our findings were statistically significant but in the lower range of clinical importance.

**Trial registration:**

The study was registered at : NCT02154620 03/06/2014 and NCT01268397 30/12/2010. Ethical approval was obtained from Ethical Committee in Stockholm, Sweden (2009/37–31/3, 2013/105–31/2, 2014/1041–32, 2017/611–32).

## Introduction

Distal radius fracture (DRF) is the most common fracture in the general population. In the aged population it is the second most common after hip fracture [1, 2]. The incidence rate of distal radius fracture increases with age [1, 3–5] and women are more vulnerable due to a high rate of osteoporosis [6, 7].

Most DRF are treated non-operatively. If the fracture is unstable, non-operative treatment often results in displacement of the fracture and malunion. Surgical treatment with open reduction and internal fixation increases the probability of fracture healing in anatomical alignment [8, 9]. In younger adults malunion results in inferior function of the wrist and hand [10–13]. This correlation is weaker in older individuals [14] and becomes even weaker with increasing age [15]. Surgery with volar locking plates has become increasingly popular even in the oldest population [3, 16–19] despite inadequate scientific support [20–25]. To the best of our knowledge there are no publications presenting comparative treatment results in the oldest population beyond 2 years after injury. A study comparing the results between 6 and 12 months in geriatric patients shows decrease in PRWE during that timeline [26] and it’s unknown if the PRWE will continue to decrease and if any benefit seen at 1 year would still be present after an extended period of time. In younger patients, continuous improvement with regards to PROM has been reported beyond 1 year [27, 28].

The aim of this study was to perform a 3 year follow-up of individuals who, at time of DRF injury, were ≥ 70 years and randomized either to surgery with volar locking or non-operative treatment with closed reduction and cast immobilization. The primary outcome was Patient Rated Wrist Evaluation (PRWE) [29] score at 3 years and secondary outcomes include additional patient reported outcome measures (PROM), range of motion (ROM), grip strength, radiographic results and complications.

## Material and methods

### Design

This study is a 3 year follow-up of a previously published randomized controlled study [30] conducted at Södersjukhuset Hospital (SH) and Danderyd Hospital (DH), both level II trauma centers in Stockholm, Sweden. Patients participating in the original study were allocated to non-operative treatment or surgery with open reduction and fixation with a volar locking plate by one to one ratio. At DH inclusion of study participants took place between December 2009 and January 2017 and at SH, April 2013 to January 2017. Initially this was two separate studies including patients ≥75 but due to a low inclusion rate and similar study protocols they were merged in 2017. The DS study had DASH as the primary outcome while SH had PRWE, but the same data was obtained. The SH study had lowered the inclusion age to 70 years to increase the inclusion rate. The collection of data for the present study was conducted August 2016 – January 2020 and took place at Södersjukhuset hospital, department of orthopedic surgery. The study ended 3 years after the last patient of the original study had been asked to participate and been evaluated.

### Participants

Patients were eligible for inclusion if they participated in the 1 year follow-up of the previously published study and 3 years had elapsed from injury. The DH patients included 2009–2011 were excluded from the study due to too long time elapse between fracture and start of the follow-up study. All eligible patients had presented to hospital with an acute dorsally displaced distal radius fracture, arbeitsgemeinschaft für osteosynthesefragen/Orthopaedic Trauma Association (AO/OTA) classification A and C with a dorsal tilt ≥20 degrees from a plane perpendicular to the length axis of the radius and met all inclusion criteria and none of the exclusion criteria listed in Table 1.

**Table 1:** Inclusion and Exclusion Criteria of a randomized controlled study comparing non-operative treatment with volar locking plate in patients > 70 years of age

**Inclusion criteria**	Patient age ≥ 75 years (≥70 years after September 2015 at SH)
	Fracture diagnosed no more than 3 days from injury at SH or 6 days at DH
	Resident of the catchment area of either study site
	Wrist radiograph of ≥20°dorsal tilt from plane perpendicular to the length axis of the radius (or ≥ 4 mm ulnar variance at DH)
**Exclusion criteria**	Associated ulna fracture
	Intraarticular displacement exceeding 1 mm step-off or 1 mm gap (SH)
	Injury to the ipsilateral upper extremity
	High energy trauma (SH)
	Former disability at either wrist
	Rheumatoid arthritis or severe joint disorders
	Dementia or Pfeffer score < 5 at SH or < 8 at DH
	Substance abuse or psychiatric disorder
	Dependency in activities of daily living
	Not fit for surgery or American Society of Anesthesiology class ≥4

### Interventions

Interventions have been described in detail in the previous publication [30]. All patients were treated with closed reduction and immobilization with a dorsal underarm splint. Patients allocated to non-operative treatment retained the splint for 4–5 weeks. Patients allocated to surgery were treated with open reduction and internal fixation with a volar locking plate. Implants used were 2.4-mm Variable Angle LCP Two-Column Volar Distal Radius Plate (DePuy Synthes), or DVR plate (ZimmerBiomet), Acu-Loc plate (Acumed) or Königsee plate (Swemac). All study participants were referred to an occupational therapist.

### Follow-up

The present study contains data from an assessment conducted at 3 years after inclusion. Close to the three-year mark of injury patients were contacted by post and over the phone and were invited to participate. Patients who could not come for investigation exactly 3 years after injury were invited to participate at an occasion convenient to the patient even if it delayed the appointment. After signing a written consent, patients filled in PROM questionnaires and underwent radiographic and clinical examinations at an outpatient visit attended by an orthopedic surgeon from the study group and an independent occupational therapist. Some patients declined to come to the hospital for examination and radiography but provided PROM results by mailing their questionnaires. The study was registered at clinicaltrail.gov (NCT02154620 and NCT01268397). Ethical approval was obtained from Ethical Committee in Stockholm, Sweden (2009/37–31/3, 2013/105–31/2, 2014/1041–32, 2017/611–32).

### Outcomes

The primary outcome of this 3 year follow-up was the score of Patient Rated Wrist evaluation (PRWE) [29, 31] 3 years after fracture. PRWE is a wrist-specific measure of function and pain where results from 15 factors translate to a continuous ordinal score ranging 0–100. Lower scores indicate less disability. The questionnaire has been validated for evaluation of distal radius fractures [29, 31–33]. Minimal clinical important difference (MCID) in PRWE-score for distal radius fractures has been suggested to be 11.5 points [34] but varies in different studies from 7.7–19 [35]. Disabilities of the Arm, Hand, and Shoulder (DASH) [36–38] was used as a secondary outcome. DASH ranges from 0 to 100 and the higher the number, the higher the disability. The MCID for DASH has been suggested to be 10 points [39]. Quality of life was assessed by EuroQol 5 dimensions 3 levels (EQ-5D 3 L) [40, 41] with the UK EQ-5D Index Tariff [41, 42]. The value 1 represents full quality of life and values closer to zero represent poorer quality of life. MCID is considered 0.074 points [43].

Measurement of grip strength and range of motion was performed by an unblinded occupational therapist. Grip strength was presented as absolute values in N/m^2^ and as percentages of the contralateral side. Grip strength was measured three consecutive times per hand using a Martin vigorimeter (Sammons Preston Rolyan). Results from right-handed individuals with an injury to the dominant hand were adjusted to compensate for 10% greater strength compared to the non-dominant hand [44]. Unadjusted percentages are also presented [45]. A goniometer was used to measure range of motion.

Radiographic evaluation of anteroposterior and lateral radiograph was performed by an experienced surgeon specialized in orthopedic and hand surgery (MW). Ulnar variance, dorsal tilt (presented as dorsal angulation from a plane perpendicular to the length axis of the radius), radial inclination and scapholunar angle were measured and presented in millimeters and degrees. Osteoarthritis in the radiocarpal and distal radioulnar joint was classified according to Knirk and Jupiter [46]. The presence of ulnar impaction was assessed and was defined as a radiographic conflict between the distal ulna and triquetrum or lunate in combination with signs of subchondral affection. Osteoarthritis and ulnar impaction were presented as the proportion of patients with positive radiological findings.

Complications were registered by reading medical charts and by an interview and clinical investigation of the patient. Assessment of medical charts was performed for patients who declined coming to the hospital.

Every participant was asked to score their overall impression of their wrist function 0–100 where 0 is worst possible and 100 is level of function before the injury. They were also asked whether they would recommend the treatment they were given to a friend in a similar situation.

### Sample size

Sampling in this study was a convenience sampling from the original study cohort representing all available patients for which approximately 3 years had elapsed from injury.

The original sample size was set to 120 patients (SH) and 130 (DH), to allow for an estimated 20% (SH) and 25% (DH) drop out and to have 90% power to detect a difference of 10 points in PRWE with a significance level set to 0.05.

### Randomization and blinding

The patients were randomized 1:1 to either treatment using randomly ordered sealed opaque envelopes. No stratification was used. Patients and researchers were not blinded to treatment. Research team and research nurse enrolled the patients and assigned patents to interventions.

### Statistical methods

Intention to treat analyses were performed for all outcomes, meaning that analysis was conducted on the data from available patients as they were randomized. Most variables were not normally distributed and were therefore presented as medians with corresponding interquartile range (IQR). Means, 95% confidence intervals and ranges were also presented. Mann-Whitney U test was used to compare numerical values between groups. When the samples were related, Wilcoxon signed rank test was used. Categorical variables were presented as numbers and proportions expressed as percentages. Two-sided Fishers exact test and Chi-square test were used for group comparisons. Mixed models were used to analyze difference over time and interaction between time and treatment. Correlation between numerical values was investigated by scatterplots and ANOVA regression. SPSS Statistics 25 (IBM) was used for statistical analysis.

## Results

**Fig. 1:**
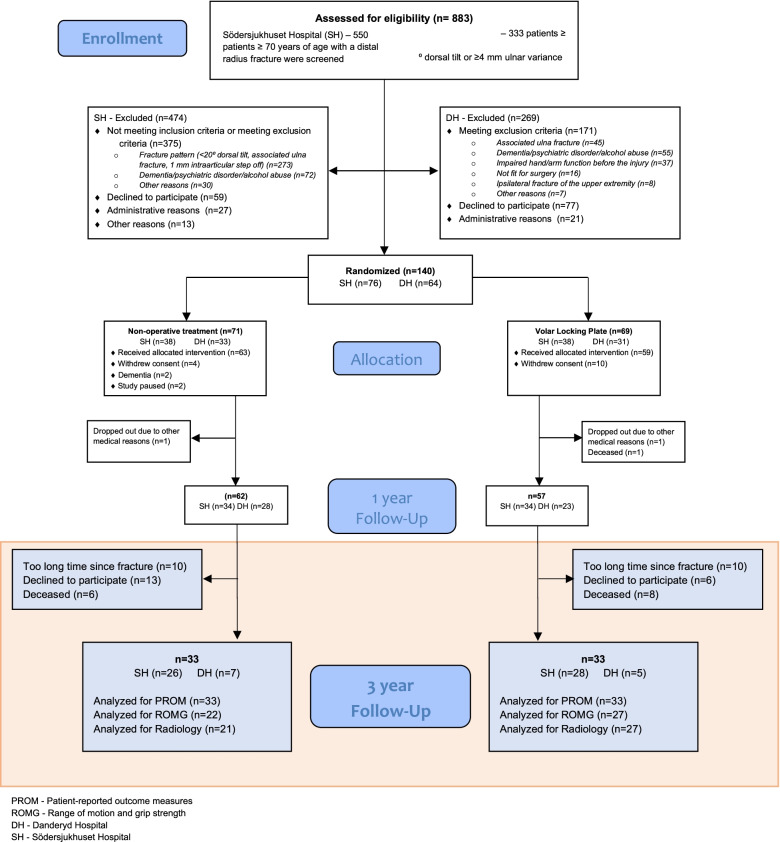
CONSORT flow diagram of an RCT comparing treatments for dorsally displaced distal radius fractures

Out of 119 eligible study persons 66 patients were analyzed for the primary outcome (Fig. 1): 33 in the non-operative treatment group and 33 who were treated with a volar locking plate. Some patients were deceased, and some declined to participate for other reasons (Fig. 1). The baseline characteristics were similar between groups (Table 2). The patients available for follow-up after 3 years were younger than the patients not available. The distribution of gender and fracture characteristics did not differ between patients randomized for the one-year follow-up and the patients analyzed in the present study (Fishers Exact Test). The PRWE and DASH mean and median scores at the 1 year follow up did not differ between individuals participating in the 3 year follow-up and the individuals who did not participate, nor from the total population participating in the one-year follow-up (Mann-Whitney U-test). If there were any unmeasured differences between the groups remain unknown. The mean time for follow-up was 38 months, range 33–52 months.

**Table 2:** A presentation of demographic and radiologic parameters in patients fulfilling the one- and three-year follow-up of a randomized controlled study comparing non-operative treatment (non-op) with volar locking plate (VLP) in patients > 70 years of age

	Available at follow-up at 3 years (*n* = 66)		Not available at follow-up at 3 years (*n* = 54)	
	Non-op *n* = 33	VLP *n* = 33	Non-op (*n* = 30)	VLP (*n* = 24)
Median age at injury (range)	76 (70–89)	78 (70–90)	80 (71–98)	81 (75–89)
Female sex, n (%)	27 (82%)	32 (97%)	28 (93%)	22 (92%)
Injury to dominant hand, n (%)	11 (33%)	15 (45%)	11 (37%)	11 (46%)
AO/OTA-classification, n (%)				
A2	4 (12%)	3 (9%)	5 (17%)	4 (17%)
A3	13 (39%)	19 (58%)	14 (47%)	13 (54%)
B1	0	0	0	1 (4%)
B2	0	0	2 (7%)	0
B3	0	0	2 (7%)	1 (4%)
C1	13 (39%)	7 (21%)	4 (13%)	2 (8%)
C2	3 (9%)	3 (9%)	3 (10%)	3 (13%)
C3	0	1 (3%)	0	0
Dorsal displacement at primary x-ray, degrees mean ± SD (median;IQR)	27 ± 7 (27;12)	30 ± 12 (30;18)	27 ± 9 (27;9)	26 ± 12 (26;18)
PRWE at 12 months mean ± SD (median;QR)	22 ± 22 (15;39)	15 ± 16 (9;23)	23 ± 22 (16;34)	11 ± 13 (6;17)

*n* numbers, *AO/OTA* arbeitsgemeinschaft für osteosynthesefragen/Orthopaedic Trauma Association, *SD* standard deviation, *IQR* inter quartile range

### Patient reported outcome measures

**Fig. 2:**
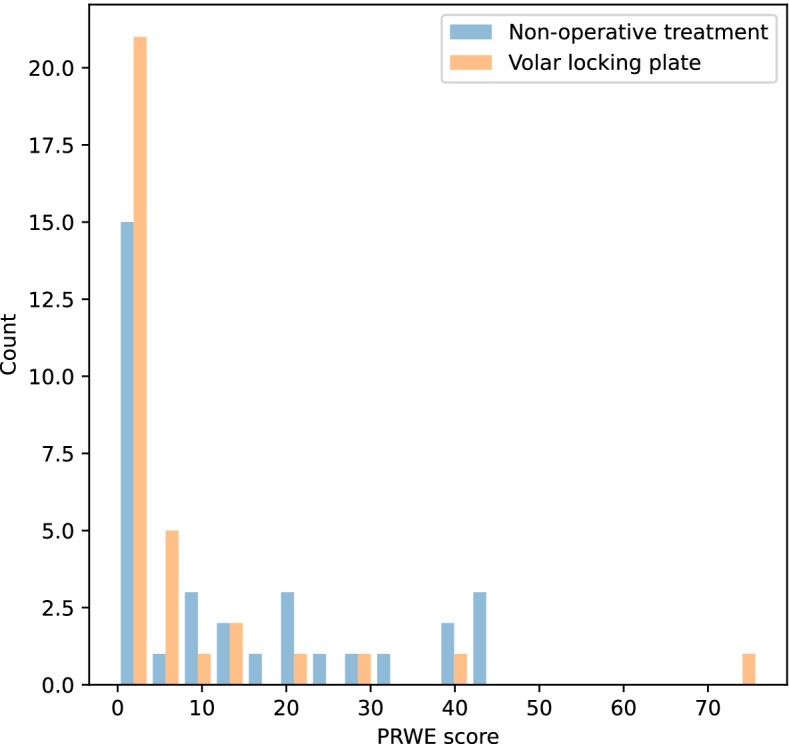
Histogram of PRWE (Patient Rated Wrist Evaluation) 3 years after a distal radius fracture in patients ≥70 years in a randomized to non-operative treatment or surgery with volar locking plate

The median PRWE was lower (representing lower disability) in the volar locking plate group (0 points) compared to the non-operatively treated group (9 points) (*p* = 0.027, Mann-Whitney U-test), Fig. 2, Table 3. In DASH scores and EQ-5D, no statistically significant difference was found.

**Table 3:** Patient Reported Outcome Measures in patients included in the 3 year follow-up of a randomized controlled study comparing non-operative treatment with volar locking plate in patients > 70 years of age

Outcome	Non-operative treatment N = 33	Volar Locking Plate N = 33	*p*-value
			Mann-Whitney U-test
PRWE score (points)			
Mean (95% CI)	13 (8–19)	7 (2–12)	
Median (IQR)	9 (23)	0 (7)	0.027
Range	0–46	0–76	
DASH score (points)			
Mean (95% CI)	15 (10–21)	11 (5–16)	
Median (IQR)	10 (18)	3 (16)	0.065
Range	0–57	0–63	
EQ5D score (points)			
Mean (95% CI)	0.85(0.79–0.91)	0.80(0.72–0.88)	
Median (IQR)	0.80(0.24)	0.80(0.31)	0.311
Range	0.19–1	−0.02-1	

*CI* confidence interval, *IQR* interquartile range

**Fig. 3:**
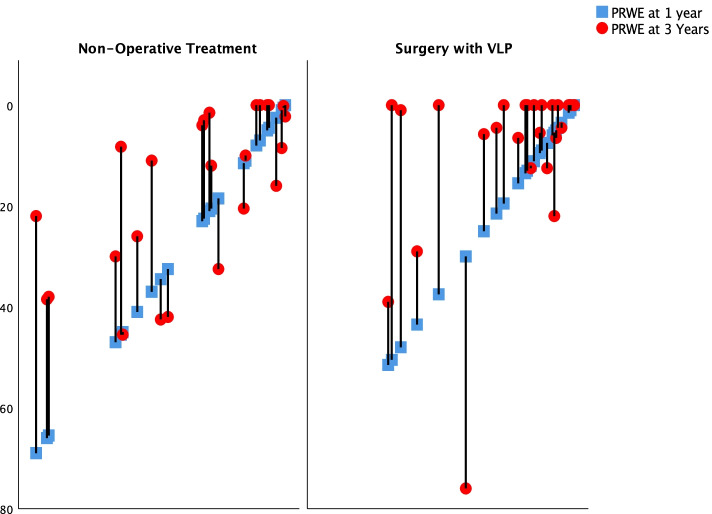
Sixty-six patients over the age of 70 sustained a dorsally displaced distal radius and were allocated to surgery with a volar plate or non-operative treatment. The diagram illustrates the change in Patient Rated Wrist Evaluation (PRWE) for each individual between the 1 year (blue square) and 3 year follow-up (red circle). The patients are displayed according to worst to best wrist-function (left to right) at 1 year

As a secondary outcome the differences between 1 and 3 years were explored. There was no significant difference in mean differences between one and 3 years between the treatment groups 95% CI (− 8) – (+ 9) (t-test). There was, however, a decrease in PRWE within each group between the 1 and 3 year follow-up (Fig. 3). In the VLP group the median PRWE decreased from 9 to 0 (*p* = 0.004) and in the non-operatively treated from 15 to 9 (*p* = 0.02) (Mann-Whitney U-test) representing a mean difference of − 7 (95% CI − 1 to − 14) for the VLP group versus − 8 (95% CI − 2 to − 14) for the non-operative group (t-test).

### Range of motion and grip strength

Forty nine patients were clinically examined for range of motion (ROM) and grip strength (Table 4). There was no significant difference in grip strength between the groups (Mann-Whitney U-test). The group treated with volar locking plate had more volar flexion and, when compared to the non-injured side, a larger ROM in supination. Other ROM measurements were similar between groups. When comparing the total ROM with the contralateral hand there was a difference in favor of the volar locking plate group (Mann-Whitney U-test).

**Table 4:** Range of motion and grip strength in patients included in the 3 years follow-up of a randomized controlled study comparing non-operative treatment with volar locking plate in patients > 70 years of age

	Non-operative treatment (*n* = 22)					Volar Locking Plate (*n* = 27)					
	Mean	SD	95% CI	Median	IQR	Mean	SD	95% CI	Median	IQR	*p*-value*
Adjusted grip strength (%)	97	20	89–106	93	17	100	17	93–107	104	27	0.191
Grip strength %	95	20	86–104	88	23	101	17	94–107	98	23	0.095
Grip strength injured hand (N)	22	9	18–25	20	9	20	4	19–22	19	5	0.904
Dorsal extension (%)	93	11	88–98	100	14	91	11	87–96	95	17	0.585
Dorsal extension (°)	56	8	52–59	56	11	54	8	50–57	55	10	0.465
Volar flexion (%)	81	24	71–91	76	34	96	25	86–106	100	11	0.005
Volar flexion (°)	61	15	54–68	60	21	68	17	61–75	72	20	0.022
Flexion-extension arc range (%)	86	14	79–92	86	20	93	14	87–99	95	14	0.015
Flexion-extension arc range (°)	117	19	108–125	114	29	122	19	115–129	126	22	0.150
Ulnar deviation (%)	97	17	89–104	100	0	105	15	100–112	100	9	0.192
Ulnar deviation (°)	26	5	24–29	30	9	29	5	27–31	30	5	0.157
Radial deviation (%)	93	12	88–98	100	13	99	19	92–107	100	9	0.224
Radial deviation (°)	24	5	22–27	25	10	23	4	22–25	23	5	0.437
Radial-ulnar deviation arc (%)	95	13	89–100	100	10	99	11	95–103	100	9	0.244
Radial-ulnar deviation arc (°)	51	9	47–55	53	10	52	6	50–55	50	9	0.824
Supination (%)	90	12	84–95	90	19	98	9	94–101	100	7	0.007
Supination (°)	102	15	70–120	110	21	109	15	104–115	110	16	0.100
Pronation (%)	95	6	93–98	100	11	98	9	94–101	100	0	0.073
Pronation (°)	86	8	83–90	87	10	88	9	85–92	90	7	0.130
Total rotation range (%)	92	8	88–96	93	15	98	6	95–100	93	15	0.016
Total rotation range (°)	188	21	179–197	197	34	198	20	190–206	205	30	0.107
Total ROM (%)	90	9	86–94	89	13	96	7	94–99	98	5	0.004
Total ROM (°)	356	43	337–375	353	74	372	32	359–384	370	58	0.157

Values in actual degrees (°) or in percentages of the contralateral side (%)

*Mann-Whitney U Test

Adjusted for dominant right hand by reducing the value to 0.9091 times the measured value

*ROM* Range of motion, *CI* confidence interval, *N* newton/m2, *n* numbers

**Fig. 4:**
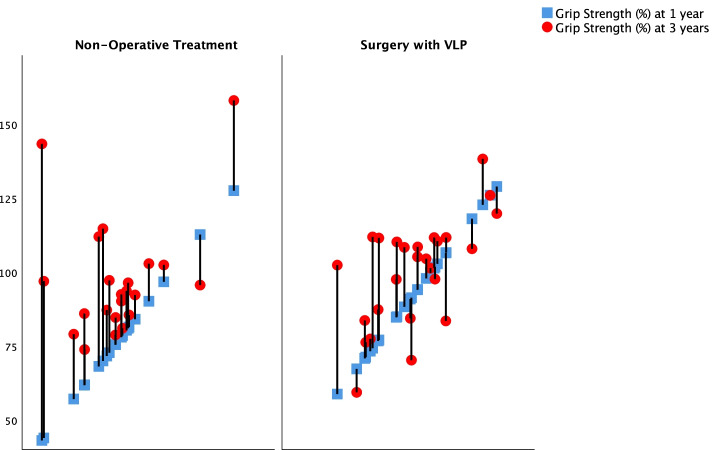
Sixty-six patients over the age of 70 sustained a dorsally displaced distal radius and were allocated to surgery with a volar plate or non-operative treatment. The diagram illustrates the change in Corrected grip strength as a percent of the strength of the non-injured hand for each individual between the 1 year (blue square) and 3 year follow-up (red circle). The patients are displayed according to worst to best corrected grip strength (left to right) at 1 year

There was no statistically significant difference in the increase of corrected grip strength in percent of the non-injured hand over time between treatments (*p* = 0.068) using a mixed models analysis, with allocation and time as main effects and allocation combined with time as the interaction. An overall increase in grip strength not taking allocation under consideration was confirmed (*p* < 0.001). Within the non-operative treated group there was a statistically significant increase of median from 77 to 93% (*p* < 0.001). Within the VLP-group the increase from 93 to 105% was non-significant (*p* = 0.25) (Wilcoxon signed rank test) (Fig. 4).

### Radiographic outcome

There was no statistically significant difference in the prevalence of osteoarthritis (Table 5) (Fishers Exact test). The nonoperatively treated group had statistically significant greater dorsal tilt and ulnar variance, and less radial inclination (Table 6) (Mann-Whitney U-test). When performing a post-hoc ANOVA investigating dorsal angulation and PRWE, there was a statistically significant small correlation (*r*^2^ = 0.13) between larger dorsal tilt and lower PRWE outcome (*p* = 0.011).

**Table 5:** Radiographic outcome in patients included in the 3 year follow-up of a randomized controlled study comparing non-operative treatment with volar locking plate in patients ≥70 years of age

	Non-operative treatment (*n* = 21)	Volar Locking Plate (n = 27)	*P*-Value
Radiocarpal osteoarthritis			0.738*
No osteoarthritis	14 (67%)	20 (77%)	
Grade 1	7 (33%)	6 (23%)	
Grade 2	0 (0%)	0 (0%)	
Grade 3	0 (0%)	0 (0%)	
Ulnar Impaction	9 (40.9%)	5 (19.2%)	0.122*
Distal ulnar radial joint osteoarthritis	2(9.5%)	3 (11.5%)	1.000*
DISI	5 (25%)	5 (19.2%)	0.726*

Radiocarpal osteoarthritis according to Knirk and Jupiter,

* Fishers exact test

*DISI* Dorsal intercalated segment instability, Scapholunate angle > 80°

n = numbers

**Table 6:** Radiographic outcome in patients included in the 3 years follow-up of a randomized controlled study comparing non-operative treatment with volar locking plate in patients > 70 years of age

	Non-operative treatment (*n* = 21)					Volar Locking Plate (*n* = 27)					*P*-value*
	Mean	Std D	95% CI	Median	IQR	Mean	Std D	95% CI	Median	IQR	
Dorsal tilt (°)	11	10	6–16	15	20	−3	8	(−6)-1	−5	12	< 0.001
Radial Inclination (°)	16	6	13–18	15	5	21	4	20–23	21	6	< 0.001
Ulnar variance (mm)	3	2	2–4	3	4	0.8	2	0–2	0	2	0.003
Scapholunar angulation (°)	71	10	67–75	70	14	67	9	63–70	69	13	0.272

n: numbers

°: degrees

*Std D* Standard deviation, *CI* confidence interval for the mean

* Mann-Whitney u-test

### Complications

All complications are presented in Table 7. At the 3 year follow-up, eight of the 66 study patients had undergone additional surgery in the wrist or hand: 5 (15%) of the non-operatively treated and three (9%) of the surgically treated patients. Only one patient had a reoperation performed between the 1 year and the 3 year follow-up (a correction osteotomy in the non-operatively treated group).

**Table 7:** Complications in patients included in a 3 year follow-up of a randomized controlled study comparing non-operative treatment with volar locking plate (VLP) in patients > 70 years of age

**Major complications**		
	Non-operative treatment (*n* = 33)	Volar Locking Plate (*n* = 33)
Additional surgery with VLP due to unacceptable secondary displacement	1	0
Corrective osteotomy	2	0
Plate extraction and carpal tunnel release	0	1
Plate extraction	0	1
Carpal tunnel release	2	1
Total	5 (15%)	3 (9%)
**Minor complications**		
	Non-operative treatment (*n* = 24)	Volar locking plate (*n* = 28)
Numbness	1	5
Scar adherence	0	1
Ulnar pain	4	1
Total	5 (21%)	7 (25%)

*n* numbers

### Other

When patients were asked to rate their wrist on an overall 100-point scale, the VLP group rated their overall wrist function higher, with a median of 99 points (IQR 4), while the non-operative treated group rated their wrist at 80 points (IQR 25). This difference was statistically significant (*p*-value = 0.001) (Mann-Whitney U-test). 75% of patients treated non-operatively would recommend the treatment to a friend compared to 100% in the volar locking plate group (*p* = 0.007) (Fishers exact test).

## Discussion

This 3 year follow-up of a randomized clinical trial of dorsally displaced distal radius fractures in a population over the age of 70 provided evidence of a benefit of surgical treatment with volar locking plate over non-operative treatment with respect to patient reported wrist function as measured by PRWE.

Our finding of a lower PRWE in the non-operatively treated group 3 years after treatment was statistically significant. The difference was present already at 1 year and did not change significantly between one and 3 years. The difference in PRWE medians between groups was 9 points and thus in the lower range of what has been suggested as the threshold for clinical significance that has been reported to vary between 7.7 and 19 [35]. In the context of a questionnaire outcome this might be due to a floor effect, where a large portion of the responders choose the lowest possible number in estimated disability. Floor values may be considered to reveal insufficient measuring qualities of the instrument used. It may also illustrate an acceptance of lost function, or rather a new idea of normal developing after 3 years where patients no longer perceive an acquired disability. Up to 9 months after an injury no flooring effects have been reported for the PRWE [47]. However, in this material, 3 years after injury, we had a considerable flooring effect, with a total of 46% of patients scoring 0 points and 67% of patients scored below 10 points. The low points we measured could clearly affect the MCID. We speculate that one cannot assume linearity of MCID along all values of PRWE, and different levels of MCID are probably relevant at different stages of recovery. The distribution of PRWE was skewed, which is a common feature for PROM results at long term follow up regardless diagnosis. Moreover, although a numerical value, PROM values cannot be considered an interval scale measurement. For these reasons non-parametric test of the medians was used for comparisons. Testing differences using the PROM means by parametric analysis would violate the assumption of normality.

This study was designed to evaluate potential differences in PRWE at 3 years after a distal radius fracture. Our choice of a region specific PROM as primary outcome, containing a pain scale and measuring function, is supported by other authors [48]. Many of our secondary outcome measures did not reveal significant differences between treatments 3 years after injury. All secondary outcomes in our study should be interpretated with caution firstly due to lack of power and secondly due to risk of random findings due to multiple sampling caused by the amount of outcome measures.

There was no difference in decrease rate in PRWE from 1 to 3 years between treatments but both groups showed improvement in PRWE, DASH as well as in grip strength between one and 3 years. The 95% CIs of mean differences for PRWE and DASH both involve MCID levels. It may be reassuring for patients to be informed that they can still expect improvement after 1 year.

Randomized trials similar to ours comparing non-operative treatment with volar locking plates in elderly patients have been carried out in the last decade but few studies present data beyond 1 year of follow-up. In contrast to our findings, most authors do not report any significant benefit of surgical treatment [9, 20, 22, 23, 25]. The inclusion criteria however differ between studies in both age range and degree of fracture displacement, making comparisons complicated. Arora et al. present no differences between groups at 12 months, but the fractures in their study healed in nearly anatomical positions, even in the non-operative group [20]. Lawson et al. showed no benefit as measured in PRWE in the RCT with patients > 60 years including fractures with more than 10-degrees of dorsal displacement [22]. At 12-months follow-up Egol et al. did not find surgery to be superior according to DASH, in a case control study of patients aged > 65 years. Their inclusion criteria may include patients with a less pronounced displacement [9]. Hassellund et al. designed a non-inferiority RCT including patients > 65 years with dorsal displacement of > 10 degrees. Non-operative treatment was not found to be inferior to surgical treatment according to quickDASH 12 months after fracture [23]. In our study we only included fractures dorsally displaced more than 20 degrees from a plane perpendicular to the length axis of the radius. We present an advantage with surgery valid for patients with severely displaced fractures, whereas other authors present results for mildly displaced fractures in which surgical treatment is not needed to achieve a good clinical outcome. Our finding of a correlation between increasing dorsal angulation and worse PRWE scores supports that opinion.

Our results, showing the benefits of surgical treatment in the long-term, are supported by Martinez-Mendez et al. [49] who investigated patients 60 years or older at least 24 months after injury and showed a superior outcome in the surgically treated group. Their radiologic inclusion criteria of initial displacement were not defined, but all included patients had a dorsal angulation of less than 15 degrees after closed reduction.

Even though many studies have been conducted investigating treatment of distal radius fractures in the aged population consensus on how these patients should be treated is still lacking. It could indicate that chronological age alone may not be sufficient for decision-making regarding treatment for displaced distal radius fractures. We speculate that other patient factors are more important. One factor that could be useful for treatment choice could be to judge functional levels in patients, as has been suggested by the Swedish guidelines for the management of distal radius fractures [50, 51].

According to our analysis of postoperative complications one correction osteotomy due to severe malunion with pain and loss of function was the only major complication occurring between 1 and 3 years after treatment. No flexor tendon ruptures were found in our material. Tendon injuries maybe expected to occur more often after volar plating in an ageing population as compared to younger individuals where tendon ruptures have been reported in 1–3% of patients surgically treated with a VLP [52–54]. However, the mean age of patients with complications has in fact shown to be lower than the mean age of patients without complications [52]. Generally, these complications are rare, and our study was not designed nor powered to detect and quantify rare complications. Future studies are needed to evaluate complication burdens after surgical treatment of older patients.

### Strengths and limitations

In this study 3 years after injury, there was a significant loss to follow-up. Only 66 of eligible 119 patients were able to participate and thus our pre-determined sample-size calculations that were used for the one-year follow-up was not met at 3 years. This large study drop-out could introduce confounding factors earlier neutralized by randomization. Even though our presentation of demographic and radiographic features reveals no differences between our follow-up patient sample and the original cohort, it is unknown whether new selection bias was introduced by patients choosing not to participate. All cohorts of the age-group under study are challenging for long term follow-up. Some patients died and some patients were unable to complete the forms and make the trip to the hospital for examination. Baseline PROM data are missing, limiting the possibility to address missing data and confounding or preform sensitivity analysis. The injury had already happened when the patients were included and the attempt to collect PROM as before resulted in unreliable data. The lack of blinding is another limitation that could affected the patient’s perception of the result. This study included many secondary outcomes and in situations with multiple testing there is always a risk of finding differences by chance. The major strength of this study is its unique presentation of 3 year follow-up data of a randomized cohort of distal radius fracture patients in aged individuals (mean age at follow-up of 80 years). Previous publications encompass only 1 year of follow-up.

## Conclusion

This study shows small but statistically significant less disability after surgery with volar locking plate compared to non-operative treatment with regards to PRWE 3 years after a dorsally displaced distal radius fracture in a population over the age of 70.

## Data Availability

The datasets generated and analysed during the current study are not publicly available due to limitations in the ethical approval but are available from the corresponding author on reasonable request.

## Abbreviations

AO/OTA: Arbeitsgemeinschaft für osteosynthesefragen/Orthopaedic Trauma Association
EQ-5D 3 L: EuroQol 5 dimensions 3 levels
DH: Danderyd Hospital
DRF: Distal radius fracture
IQR: interquartile range
MCID: Minimal Clinical Important Difference
RCT: Randomized clinical trial
ROM: Range of motion
PROM: Patient reported outcome measures
PRWE: Patient Rated Wrist Evaluation
SH: Södersjukhuset Hospital
VLP: volar locking plate
